# Health Insurance, Poverty, and Medical Costs Before and After Disability Application

**DOI:** 10.1093/geroni/igaf122.1519

**Published:** 2025-12-31

**Authors:** Jennifer Kaufman, Jasmine Manalel, Jenna Tipaldo, Na Yin, Ruth Finkelstein

**Affiliations:** Brookdale Center for Healthy Aging at Hunter College, New York, New York, United States; Hunter College, CUNY, New York, North Carolina, United States; City University of New York, New York, New York, United States; Baruch College, CUNY, New York City, New York, United States; Hunter College, New York, New York, United States

## Abstract

Social Security Disability Insurance (SSDI) and Supplemental Security Insurance (SSI) programs are designed to provide a safety net after disability onset. SSDI qualifies an individual for Medicare after a two-year waiting period, and SSI qualifies an individual for Medicaid almost immediately. Yet applying for benefits is a lengthy process. Disabled workers lacking health insurance while they wait may experience deteriorating health and financial insecurity, compounding economic and health disparities. To learn how health insurance, financial security, and out-of-pocket costs changed from before disability application to the years after, we analyzed disabled workers aged 51-64 using data from the HRS linked to Social Security administrative records. Our findings show that disability benefits are significantly associated with higher rates of health insurance coverage. By the second wave (two to four years) after the disability decision, those with SSI and Medicaid eligibility had lower out-of-pocket costs than those rejected for benefits. For the group awarded SSDI only, out-of-pocket costs were higher at baseline, spiked at the wave (within two years) following the decision, and remained substantially higher than for the other groups. Poverty rates were markedly different between groups at baseline and showed no significant change across waves. Our findings confirm that disability benefits generally work as intended to provide a safety net; Medicaid eligibility is particularly important. Yet many adults with disabilities experience poverty as they approach old age. Future research should examine longer-term outcomes and whether transitioning into Medicare evens things out for those who do not receive disability benefits.

